# Lanolin and Vaseline

**Published:** 1891-01-24

**Authors:** 


### LANOLIN AND VASELINE.

Mr. Luff, from his experiments, comes to conclusions as to the absorption of drugs from ointments in which these substances are employed as vehicles in place of lard, diametrically opposed to those of other observers. He asserts that the absorption of iodide of potassium, phenol and resorcine was far more rapid from vaseline than from lard, and that from lanolin it was nil after twenty-four hours. Previous experiments made with atropine, the absorption of which is early and positively indicated by the dilatation of the pupil, had given the place of honour to lanolin, and considering that lanolin is the natural fatty secretion of the skin, while vaseline is properly not a fat at all, this estimate of their relative advantages seems *à priori* the more probable. The question calls for further investigation, but we must confess that we shall require strong evidence before we can accept Mr. Luff's startling conclusions.

